# Enhanced Fear Memories and Altered Brain Glucose Metabolism (^18^F-FDG-PET) following Subanesthetic Intravenous Ketamine Infusion in Female Sprague–Dawley Rats

**DOI:** 10.3390/ijms23031922

**Published:** 2022-02-08

**Authors:** Kennett D. Radford, Rina Y. Berman, Shalini Jaiswal, Sharon Y. Kim, Michael Zhang, Haley F. Spencer, Kwang H. Choi

**Affiliations:** 1 Daniel K. Inouye Graduate School of Nursing, Uniformed Services University, Bethesda, MD 20814, USA; kennett.radford@usuhs.edu; 2 Center for the Study of Traumatic Stress, Uniformed Services University, Bethesda, MD 20814, USA; rina.berman.ctr@usuhs.edu (R.Y.B.); mikezhang9416@gmail.com (M.Z.); 3 Biomedical Research Imaging Core (BRIC), Department of Radiology and Radiological Sciences, Uniformed Services University, Bethesda, MD 20814, USA; shalini.jaiswal.ctr@usuhs.edu; 4 Program in Neuroscience, Uniformed Services University, Bethesda, MD 20814, USA; sharon.kim@usuhs.edu (S.Y.K.); haley.spencer.ctr@usuhs.edu (H.F.S.); 5 Department of Psychiatry, F. E. Hébert School of Medicine, Uniformed Services University, Bethesda, MD 20814, USA

**Keywords:** intravenous ketamine, fear memory, brain imaging, sex differences, stress hormone, post-traumatic stress disorder (PTSD)

## Abstract

Although women and men are equally likely to receive ketamine following traumatic injury, little is known regarding sex-related differences in the impact of ketamine on traumatic memory. We previously reported that subanesthetic doses of an intravenous (IV) ketamine infusion following fear conditioning impaired fear extinction and altered regional brain glucose metabolism (BGluM) in male rats. Here, we investigated the effects of IV ketamine infusion on fear memory, stress hormone levels, and BGluM in female rats. Adult female Sprague–Dawley rats received a single IV ketamine infusion (0, 2, 10, or 20 mg/kg, over a 2-h period) following auditory fear conditioning (three pairings of tone and footshock). Levels of plasma stress hormones, corticosterone (CORT) and progesterone, were measured after the ketamine infusion. Two days after ketamine infusion, fear memory retrieval, extinction, and renewal were tested over a three-day period. The effects of IV ketamine infusion on BGluM were determined using ^18^F-fluoro-deoxyglucose positron emission tomography (^18^F-FDG-PET) and computed tomography (CT). The 2 and 10 mg/kg ketamine infusions reduced locomotor activity, while 20 mg/kg infusion produced reduction (first hour) followed by stimulation (second hour) of activity. The 10 and 20 mg/kg ketamine infusions significantly elevated plasma CORT and progesterone levels. All three doses enhanced fear memory retrieval, impaired fear extinction, and enhanced cued fear renewal in female rats. Ketamine infusion produced dose-dependent effects on BGluM in fear- and stress-sensitive brain regions of female rats. The current findings indicate that subanesthetic doses of IV ketamine produce robust effects on the hypothalamic–pituitary–adrenal (HPA) axis and brain energy utilization that may contribute to enhanced fear memory observed in female rats.

## 1. Introduction

Although women and men are equally likely to receive ketamine following traumatic injury, little is known regarding sex-related differences in the effects of ketamine on trauma-related outcome measures. Ketamine is a favorable trauma analgesic and anesthetic agent due to its hemodynamic stability and preservation of respiration [1]. However, at higher analgesic doses, ketamine produces transient psychoses, including hallucination, delusions, and dissociation [2,3]. These effects may lead to exaggerated fear memory consolidation if ketamine is administered during the peri-trauma period. Abnormal fear memories that fail to extinguish after a traumatic event may contribute to the development of stress-related disorders such as post-traumatic stress disorder (PTSD) [4]. Potential sex-related differences in the effects of ketamine on development of PTSD are of particular concern, since women are twice more likely to develop PTSD after a traumatic event than men, due to risk factors such as more negative appraisal of past events [5,6]. To our knowledge, there are no preclinical studies investigating the sex-related effects of intravenous ketamine infusion on PTSD-like behavior in rodents. 

Preclinical studies describing the effects of ketamine on fear memory are inconsistent, due to the fact that these studies used different doses, timing, and routes of ketamine administration to animals (see [7] for a detailed review). For instance, subanesthetic doses of ketamine injected via an intraperitoneal (IP) route after fear learning produced either increased [8], decreased [9,10,11], or no change [12] in fear memory in rodents. Moreover, different routes and doses of ketamine administration can produce heterogeneous effects on rodent fear memory. It has been shown that a 10 mg/kg IV ketamine infusion over a 2-h period enhanced fear memory while an IP injection of the same dose decreased fear memory in male rats [13]. It is important to note that ketamine is mostly administered intravenously in patients and injected intraperitoneally in animals. Given that the gap between preclinical and clinical investigations includes the route and duration of ketamine administration, it is critical to consider these factors when interpreting results from ketamine studies. Thus, the current study utilized an IV ketamine infusion in awake and freely-moving rats in order to improve clinical translation.

Sex-related differences in the effects of ketamine on various behaviors have been reported previously. It has been shown that female rodents are more sensitive to the effects of ketamine, possibly due to a slower drug metabolism and, therefore, a longer half-life of ketamine compared to male counterparts [14]. For example, female rodents exhibit greater dissociative stereotypy [15], antinociception [16], and antidepressant effects [17] than males following ketamine administration. Despite these studies on sex differences, very little is known about the differential effects of ketamine on fear memory between male and female animals. One such study reported that ketamine and its metabolite (2*S*,6*S*)-HNK attenuated fear in male, but not female, mice [18]. However, that study administered ketamine one week before contextual fear conditioning as a prophylactic treatment, thus limiting the interpretation of results and comparison with other studies. 

Previous studies suggested that gonadal hormones may influence fear memory in female rodents. For instance, estrous cycle analysis revealed that female rats in high estrogen phases exhibited facilitated fear extinction [19,20,21], and similar findings were observed in humans [22,23]. Hormonal manipulation, i.e. ovariectomy (OVX) in rats or oral contraception in humans, was demonstrated to impair fear extinction [21,22]. Additionally, behavioral effects of ketamine may also vary with the estrous cycle of female rodents. A previous study has shown that estrogen levels in intact female rats affected ketamine-induced dissociative stereotypy [16]. Other studies have reported that female rodents in the proestrus phase (high estrogen levels) or supplemented with estrogen after OVX showed greater antidepressant effects of ketamine [17,24]. Given that estrogen levels may influence fear memory and ketamine’s effects on behavior, it is important to investigate effects of estrous cycle on ketamine-induced fear memory in female rats. 

Brain glucose utilization and stress hormones may serve as two potential markers and mediators of ketamine’s effects on fear memory. It has been reported that IV ketamine infusion produced region-specific effects on brain glucose metabolism (BGluM) in adult male rats, including several regions crucial to fear and stress circuitry [13]. However, to our knowledge, there is a lack of evidence on sex-related differences in BGluM following subanesthetic doses of IV ketamine infusion either in clinical or preclinical literature. Another key player in ketamine’s impact on fear memory includes stress hormones, namely corticosterone (CORT), the rodent analog to cortisol, and progesterone, the precursor to CORT, released from the adrenal glands when the hypothalamic–pituitary–adrenal (HPA) axis is activated [25,26]. Elevated CORT levels may play a significant role in strengthening the consolidation of emotional memories through stimulation of glucocorticoid receptors present in stress-sensitive brain regions such as the frontal cortex, amygdala, and hippocampus [27]. The effects of ketamine on CORT levels are inconsistent as well. For instance, ketamine administration increased CORT levels in rats [28,29,30] and cortisol levels in humans [31,32], while ketamine may reduce CORT levels in rats and mice that were pre-exposed to stressful situations [8,33]. These studies highlight the significance of the status of HPA axis (stressed vs. non-stressed) and emotional arousal on CORT levels when ketamine was administered to the animals. 

Considering the lack of information on sex-related effects of ketamine on fear memory and brain glucose utilization, the current study investigated the effects of an IV ketamine infusion on those outcome measures in adult female rats, as a follow-up to the male rat study [13]. Based on the previous study in male rats, we hypothesized that an IV ketamine infusion after fear conditioning may enhance fear memory and alter BGluM in stress-sensitive brain regions of female rats. We further hypothesized that female rats may be more responsive to the behavioral effects of ketamine as compared to male rats.

## 2. Results

**Figure 1:**
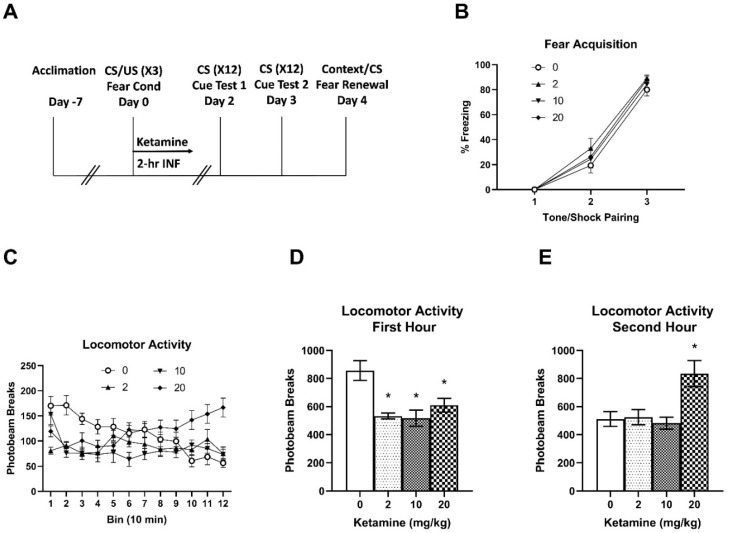
Effects of subanesthetic IV ketamine infusion on locomotor activity in female rats. (**A**) Experimental design of fear conditioning/ketamine infusion and fear memory testing. (**B**) Freezing behavior during fear conditioning (fear acquisition). Freezing gradually increased to repeated tone and footshock pairing but there were no group differences at each time point. (**C**) Time course of locomotor activity during the ketamine infusion (2 h). (**D**) Total activity during the first hour of ketamine infusion. (**E**) Total activity during the second hour of ketamine infusion. * *p* < 0.05 compared to the saline controls.

Following one week of acclimation, animals were subjected to auditory fear conditioning and ketamine infusion (Day 0). Animals acquired fear memory during the fear conditioning session (Figure 1B). A two-way RM ANOVA indicated a main effect of time (three tone/footshock pairings) F (2, 168) = 371.9, *p* < 0.001 on freezing behavior. There were no significant differences in freezing between the groups. Following fear conditioning, animals received an IV ketamine or saline infusion and spontaneous locomotor activity was monitored during the 2-h infusion (Figure 1C). A two-way RM ANOVA indicated significant main effects of ketamine F (3, 672) = 20.55, *p* < 0.001 and time F (11, 672) = 2.071, *p* = 0.02, and an interaction F (33, 672) = 4.133, *p* < 0.001 on locomotor activity. Saline controls showed a typical pattern of exploration followed by gradual habituation during the 2-h infusion period. Compared to the saline controls, all ketamine doses (2, 10, and 20 mg/kg) reduced activity during the first hour of infusion (Bins 1–6), while 20 mg/kg increased activity during the second hour (Bins 7–12) of infusion. To highlight biphasic effects of ketamine on locomotor activity, total activity for the first and second hours is presented separately. All three doses reduced activity in the first hour compared to the saline controls (Figure 1D). One-way ANOVA revealed significant effects of ketamine F (3, 56) = 8.342, *p* < 0.001 on activity. Post-hoc tests indicated that locomotor activity with all three doses was significantly different from the saline control group (*p* < 0.05). However, only 20 mg/kg ketamine increased total activity in the second hour of the infusion (Figure 1E). One-way ANOVA revealed significant effects of ketamine on total activity F (3, 56) = 6.720, *p* < 0.001. Post-hoc tests indicated that locomotor activity in the 20 mg/kg group was significantly different from all other groups (*p* < 0.05). 

**Figure 2:**
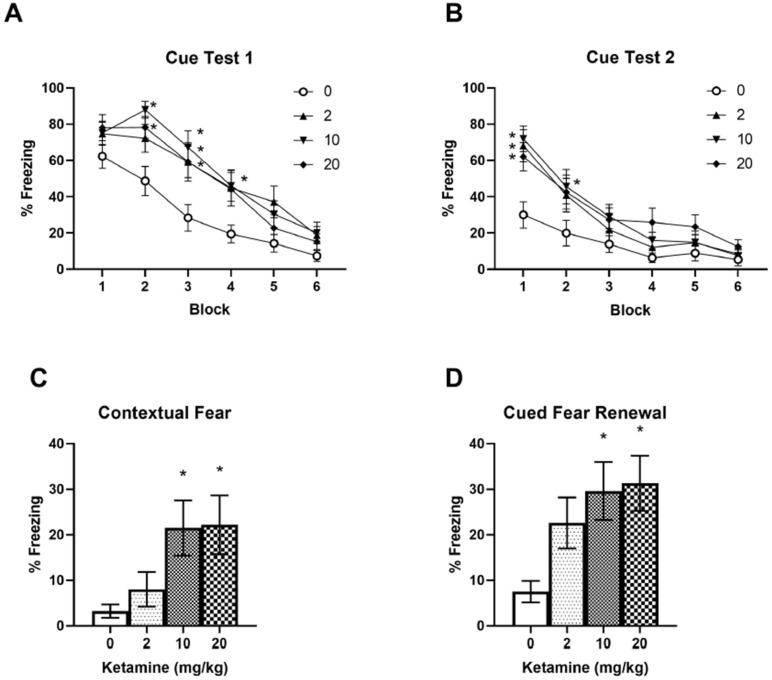
Effects of subanesthetic IV ketamine infusion on fear memory retrieval, extinction, and renewal in female rats. (**A**) Ketamine infusion increased cued fear memory retrieval with all three doses (2, 10, and 20 mg/kg, 2 h). (**B**) Ketamine infusion impaired fear extinction retrieval in female rats. Each Block consists of average freezing of two consecutive auditory tone (CS) presentations. (**C**) Higher doses of ketamine (10 and 20 mg/kg) increased contextual fear memory when animals were tested in the conditioning chamber (context A). (**D**) Higher doses of ketamine (10 and 20 mg/kg) increased cued fear memory renewal (context A) after two sessions of cued fear extinction (context B). * *p* < 0.05 compared to saline controls.

Two days after fear conditioning and ketamine infusion, fear memory extinction and extinction retrieval were tested over a two-day period (Days 2 and 3). All three ketamine doses increased initial fear memory retrieval compared to the saline controls in Cue Test 1 on Day 2 (Figure 2A). A two-way RM ANOVA indicated significant main effects of ketamine F (3, 336) = 14.73, *p* < 0.001 and time F (5, 336) = 43.40, *p* < 0.001 on freezing. Post-hoc tests revealed significant differences in freezing between ketamine (10 and 20 mg/kg) and saline groups in Block 2, and between all ketamine doses and saline in Block 3 in Cue Test 1 (*p* < 0.005), as shown in Figure 2A, indicating increased fear memory retrieval in ketamine groups. However, all ketamine groups exhibited a normal fear extinction pattern by the end of the session, as shown in Blocks 5 and 6 in Figure 2A. When animals were re-exposed to the CS the following day (Cue Test 2, Day 3) all three ketamine groups showed increased freezing compared to the saline controls (Figure 2B). A two-way ANOVA indicated main effects of ketamine F (3, 336) = 10.45, *p* < 0.001 and time F (5, 336) = 32.27, *p* < 0.001 on freezing. Post-hoc tests revealed that freezing in all three ketamine groups differed significantly from the saline control group in Block 1 of Cue Test 2 (Figure 2B). This indicates that fear extinction retrieval is impaired in ketamine groups (Cue Test 2), although these animals learned fear extinction in the previous day (Cue Test 1). However, animals in the ketamine groups eventually learned normal fear extinction during Cue Test 2, as shown from Blocks 3 to 6 in Figure 2B. These findings indicate that ketamine animals can acquire fear extinction within a session, although they are more vulnerable to relapse of fear memory triggered by the CS.

Following two days of fear extinction in Context B (Days 2 and 3), fear memory renewal was tested in Context A (fear conditioning chamber) without the US (footshock) on Day 4. Only 10 and 20 mg/kg ketamine doses increased freezing to Context A compared to the saline controls (Figure 2C). One-way ANOVA indicated significant effects of ketamine F (3, 56) = 3.871, *p* = 0.0138 on freezing to the context. Post-hoc tests revealed significant differences in context freezing between the ketamine (10 and 20 mg/kg) groups and the saline control group (*p* < 0.05). Overall freezing to the context was low, as indicated in Figure 2C (approximately 20% freezing in ketamine animals). However, when a single auditory tone (CS) was presented following the contextual fear test, ketamine (10 and 20 mg/kg) groups showed robust freezing (approximately 30%) as compared to the saline control group (approximately 5%) (Figure 2D). One-way ANOVA indicated significant effects of ketamine F (3, 56) = 4.441, *p* = 0.0072 on freezing after the CS. Post-hoc tests revealed significant differences in freezing between the ketamine (10 and 20 mg/kg) groups and the saline control group (*p* < 0.05). These results further indicate that animals that received a ketamine infusion following fear conditioning may exhibit enhanced fear memory even after two sessions of fear extinction when triggered by a fear-associated context or cue. 

**Figure 3:**
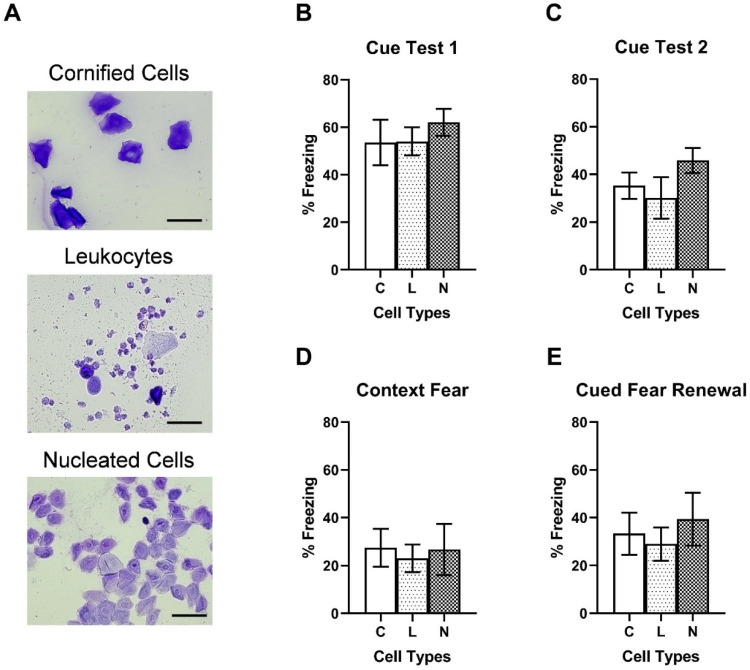
No effects of estrous cycle on ketamine-induced fear memory enhancement. (**A**) Representative images of vaginal cell types (from top to bottom): Cornified epithelial cells (C), Leukocytes (L), and Nucleated epithelial cells (N). Scale: 30 µm. (**B**) Freezing behavior during Cue Test 1. (**C**) Freezing during Cue Test 2. (**D**) Freezing behavior during contextual fear test 3. (**E**): Freezing behavior during cued fear renewal test.

In order to test the effects of estrogen levels on fear memory, female rats were assigned to three groups based on the major vaginal cell types (cornified epithelial cells, leukocytes, and nucleated epithelial cells). Because 10 and 20 mg/kg ketamine doses produced significant effects on fear memory, data from these two groups were used for vaginal cell type analysis. Representative images of the three vaginal cell types are shown in Figure 3A. Figure 3B,C shows no significant differences in freezing during the Cue Test 1 F (2, 23) = 0.363, *p* = 0.699 and Cue Test 2 F (2, 22) = 1.168, *p* = 0.329 between the groups. Additionally, freezing during the contextual fear test F (2, 19) = 0.086, *p* = 0.917 (Figure 3D) and cued fear renewal F (2, 21) = 0.3250, *p* = 0.726 (Figure 3E) was not significantly different between the groups. These results indicate that estrogen levels in intact female rats may not contribute to ketamine-induced fear memory enhancement.

**Figure 4:**
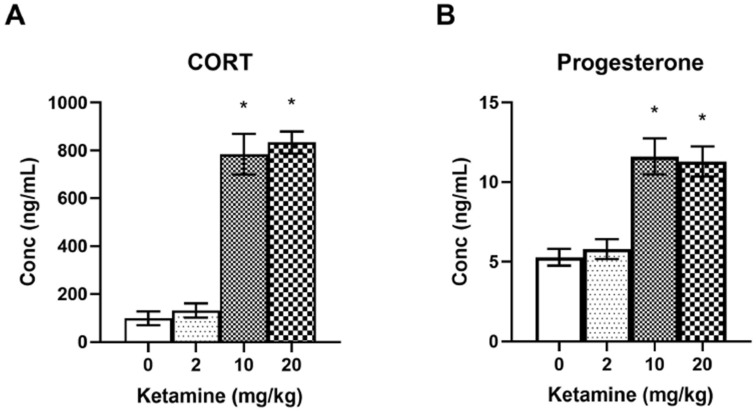
Effects of subanesthetic doses of IV ketamine infusion on plasma stress hormone (CORT and progesterone) levels in female rats. (**A**) Higher doses of ketamine (10 and 20 mg/kg) significantly elevated plasma CORT levels. (**B**) Higher doses of ketamine (10 and 20 mg/kg) also elevated plasma progesterone levels. * *p* < 0.05 compared to saline controls.

In order to determine ketamine effects on the HPA axis, plasma stress hormone levels (CORT and progesterone) were measured following a 2-h ketamine infusion in female rats (Figure 4). One-way ANOVA indicated significant effects of ketamine on CORT levels F (3, 50) = 54.58, *p* < 0.001 (Figure 4A). Post-hoc tests revealed that CORT levels in the 10 and 20 mg/kg ketamine groups were significantly elevated compared to the saline control group (*p* < 0.05). Additionally, levels of progesterone, a precursor to CORT, were also elevated following the ketamine infusion (Figure 4B). One-way ANOVA indicated significant effects of ketamine on progesterone levels F (3, 50) = 15.79, *p* < 0.001 (Figure 4B). Post-hoc tests revealed that CORT and progesterone levels in the 10 and 20 mg/kg ketamine groups were significantly elevated compared to the saline control group (*p* < 0.05). There were no differences in CORT and progesterone levels between the 10 and 20 mg/kg groups, indicating that 10 mg/kg ketamine may have produced a maximum effect on stress hormone release in female rats.

**Figure 5:**
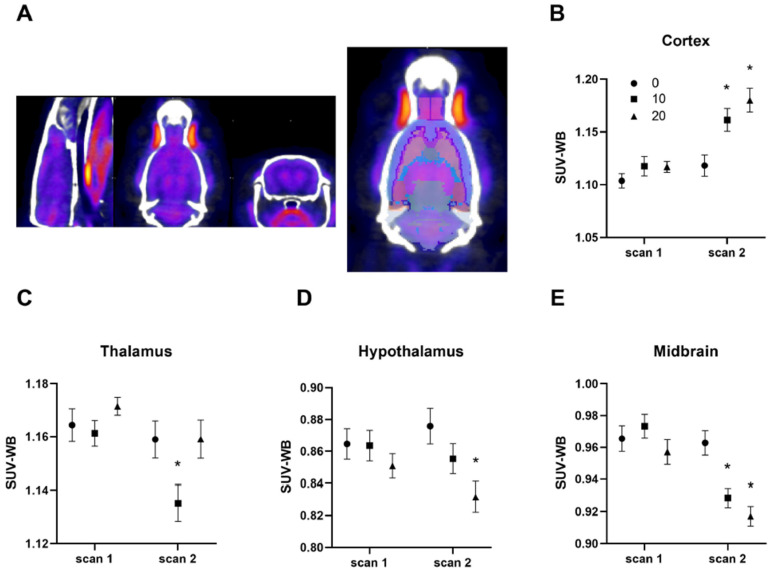
Effects of subanesthetic doses of IV ketamine infusion on BGluM of female rats. (**A**) Representative images of ^18^F-FDG-PET/CT of a rat brain. Three images including sagittal, planar, and coronal sections from left to right are shown in the first image. The second image shows a coronal section with a rat brain atlas registered to it. (**B**) Both 10 and 20 mg/kg ketamine increased BGluM in the cortex. (**C**) The 10 mg/kg dose reduced BGluM in the thalamus. (**D**) The 20 mg/kg dose reduced BGluM in the hypothalamus. (**E**) Both 10 and 20 mg/kg ketamine doses reduced BGluM in the midbrain. Baseline BGluM levels in these brain regions are not significantly different between the groups as shown in scan 1. * *p* < 0.05 compared to saline controls.

Effects of subanesthetic doses of ketamine on regional BGluM were determined using ^18^F-FDG-PET/CT imaging in female rats (Figure 5). Animals were scanned at baseline (scan 1) and immediately after fear conditioning and ketamine infusion (scan 2). None of the analyzed brain regions were significantly different between the groups at baseline (scan 1), indicating basal BGluM levels were similar between groups. The ketamine infusion produced region- and dose-specific effects on BGluM in female rats. Both the 10 and 20 mg/kg doses increased BGluM in the cortex (Figure 5A). Two-way ANOVA indicated significant main effects of ketamine, F (2, 30) = 6.567, *p* = 0.0043 time, F (1, 28) = 58.43, *p* < 0.001, and interaction F (2, 28) = 7.544, *p* = 0.0024 on BGluM. Post-hoc tests revealed that the BGluM in the cortex with 10 and 20 mg/kg doses was significantly different from the saline controls (*p* < 0.05). The 10 mg/kg dose reduced BGluM in the thalamus (Figure 5B). Two-way ANOVA indicated main effects of ketamine F (2, 30) = 3.446, *p* = 0.0449 and time F (1, 28) = 10.57, *p* = 0.003 on BGluM. Post-hoc tests revealed that BGluM in the thalamus with 10 mg/kg ketamine was significantly different compared to the saline controls (*p* < 0.05). However, the 20 mg/kg ketamine reduced BGluM in the hypothalamus (Figure 5C). Two-way ANOVA indicated a significant main effect of ketamine F (2, 30) = 3.855, *p* = 0.0324 on BGluM. Post-hoc tests revealed significant effects of the 20 mg/kg dose on BGluM in the hypothalamus compared to the saline controls (*p* < 0.05). Both 10 and 20 mg/kg doses reduced BGluM in the midbrain (Figure 5D). One-way ANOVA indicated main effects of ketamine, F (2, 30) = 5.744, *p* = 0.0077 time, F (1, 28) = 38.26, *p* < 0.001, and an interaction F (2, 28) = 7.485, *p* = 0.0025 on BGluM. Post-hoc tests revealed that BGluM in the midbrain of 10 and 20 mg/kg groups was significantly different from the saline control group (*p* < 0.005). Other brain regions investigated in the study were not statistically significant (data not shown). These findings suggest dose- and region-specific effects of IV ketamine infusions on the BGluM of female rats.

## 3. Discussion

A previous investigation found that subanesthetic doses of IV ketamine infusion following fear conditioning enhanced fear memory renewal and impaired fear extinction in adult male rats [13]. However, the effects of IV ketamine infusion on fear memory of female rats are largely unknown. Thus, the goal of the current study was to determine the effects of subanesthetic doses of IV ketamine infusion on fear memory and brain glucose utilization in adult female rats and to compare the results with the previous male rat study [13]. 

The IV ketamine infusion produced dose-dependent effects on locomotor activity of female rats. During the first hour of the ketamine infusion, all three doses (2, 10, and 20 mg/kg) suppressed locomotor activity compared to the saline controls, indicating sedative and analgesic effects of ketamine. However, only the 20 mg/kg dose stimulated locomotor activity during the second hour of infusion period. This is likely due to cumulative effects of ketamine in the body producing dissociative effects, which are more pronounced in females than males. Similarly, female rats were more responsive to the effects of IV ketamine on fear memory than were male rats in the previous study, with all three doses (2, 10, and 20 mg/kg) increasing fear memory in female rats compared to only the 10 mg/kg dose in male rats (Radford, Park et al. 2018). Females, but not males, responding to the 20 mg/kg dose of ketamine in both locomotion and fear memory tests may be attributed to slower metabolic clearance of the drug, increasing drug concentration and duration of action [14]. Similarly, female response to the 2 mg/kg ketamine dose is consistent with previous studies reporting physiological and behavioral effects of ketamine observed at lower doses for females than for males [16,17,24,34]. Specifically, female rats that received ketamine infusion showed heightened fear retrieval at the beginning of Cue Test 1 but were able to extinguish fear by the end of the session. However, when female rats returned to Context B the next day (Cue Test 2), ketamine rats showed impaired fear extinction retrieval compared to the saline controls. These findings are clearly different from male rats that only exhibited impaired fear extinction in Cue Test 1 rather than impaired fear extinction retrieval in Cue Test 2 [13]. Sex differences in fear memory learning in rodents have been reported previously, with findings that females experience more [35], less [36,37], or equal [19] fear during extinction compared to males. 

The sex differences in fear memory may be attributed to gonadal hormones as high estrogen levels were associated with facilitated fear extinction in female rodents [19,20,21]. It is important to note that the Milad et al. (2009) and Gruene et al. (2015) studies used estrous cycle data collected on the day of fear extinction learning or testing. In contrast, the current findings and other studies, which reported no significant effects of estrous cycle on rodent fear memory, used estrous cycle data collected on the day of fear conditioning [38,39,40]. Thus, the timing of estrous cycle data used for fear memory analyses may account for the discrepancy between the studies. Interestingly, different metrics of fear (i.e., freezing vs. fear-potentiated startle) may be differentially affected by the phases of estrous cycle of female rats [41]. Taken together, these studies suggest complex interaction between gonadal hormones and fear-related responses in female rodents. 

Ketamine is known to increase stress hormone levels, and this may have significant consequences on fear memory consolidation and extinction depending on the timing of ketamine administration. In numerous studies, CORT administered after fear conditioning increased the consolidation of fear memory in a dose- and time-dependent manner [42,43,44]. Previous studies reported that subanesthetic doses of IV ketamine infusion elevated plasma CORT levels in rats [29,30]. Compared to male rats [30], the same dose of IV ketamine (10 mg/kg) infusion produced an approximately two-fold higher elevation of stress hormone levels in female rats in the current study. It is well established that female rodents have a higher levels of both basal and stress-induced CORT than do males [45]. In one study, chronically stressed female rats receiving a 10 mg/kg IP ketamine injection experienced a roughly two-fold CORT elevation compared to chronically stressed male rats [28]. Interestingly, that study reported that ketamine elevated CORT levels only in conscious rats but not in isoflurane-anesthetized rats, suggesting that locomotion and behavior may contribute to the HPA axis stimulation in rats [28]. However, in the current study, both the locomotor-suppressing dose (10 mg/kg) and locomotor-stimulating dose (20 mg/kg) of IV ketamine produced elevation of plasma CORT levels (Figure 4A). Therefore, behavioral stimulation alone cannot fully explain the CORT elevation following subanesthetic doses of ketamine in rodents. Furthermore, a previous study showed no significant differences in CORT levels between fear conditioned and non-fear conditioned rats immediately after ketamine or saline infusions [29], which discounts any contribution of footshock stress to the observed CORT elevations. Therefore, the most reasonable explanation is that ketamine itself increases CORT levels, although the mechanism by which this may occur is presently unclear. 

Similar to CORT, plasma progesterone levels were also elevated following ketamine infusion in female rats. Previous studies have shown that progesterone is a precursor to CORT released by the adrenal glands in response to stress [25,26], and plasma progesterone and CORT levels are highly correlated after various stressors and HPA axis manipulations [26,46]. Additionally, OVX female rats undergoing inescapable footshocks still demonstrated a significant elevation of progesterone levels compared to non-stressed animals [47]. In OVX female rats, progesterone levels can be enhanced with ACTH and abolished with dexamethasone administration [48]. In the present study, plasma progesterone levels did not vary significantly between animals (approximately 10%); however, ketamine infusion (10 and 20 mg/kg) significantly elevated progesterone levels in these animals. There was a significant correlation between CORT and progesterone levels in the same animals. This indicates that elevated plasma progesterone levels, along with CORT levels, following IV ketamine infusion are mostly due to HPA axis stimulation rather than variations in gonadal hormones originated from the ovaries of female rats. 

Fear memory-enhancing effects of IV ketamine infusion following fear conditioning may be attributed to its stimulation of the HPA axis based on the elevation of stress hormone levels during the ketamine infusion. However, literature on the role of stress hormones on PTSD development has been controversial, with conflicting studies finding protective [49,50] or harmful [51,52] effects of stress hormones on PTSD. For instance, high-dose exogenous CORT administration produced protective effects against PTSD for individuals with impaired stress responses [49]. One consideration that needs to be taken seriously is the emotional arousal states of individuals (e.g., stressed vs. non-stressed) when ketamine is administered. This condition may produce differential, if not opposite, effects on fear- and stress-related disorders in individuals who receive ketamine. Given that female rats exhibit greater reactivity to ketamine-induced CORT elevation and fear memory consolidation, the current findings shed light on the significance of biological mechanisms underlying sexual dimorphism of PTSD. Furthermore, this information may inform clinicians on how to treat wounded male and female patients properly when utilizing IV ketamine infusion as a trauma analgesic drug. 

Administering a drug with dissociative properties, such as ketamine, after emotional learning may paradoxically enhance memory consolidation. There are several studies suggesting retrograde facilitation, the enhancement of memory encoded prior to drug administration, caused by various drugs such as alcohol, midazolam, and propofol [53,54,55]. Thus, it is possible that ketamine may also produce similar effects on fear memory consolidation when given immediately after fear learning. This presents an interesting hypothesis that ketamine may enhance fear memory consolidation as well as fear extinction depending on the timing of drug administration. Thus, future studies are warranted to determine the mechanisms of IV ketamine infusion on different aspects of fear memories in the context of cognitive and behavioral intervention combined with ketamine administration.

In addition to stress hormone levels, in vivo brain glucose utilization can be a useful measure following IV ketamine infusion. In the current study, IV ketamine infusion produced dose-dependent effects on BGluM in several brain regions including the cortex, midbrain, hypothalamus, and thalamus of female rats. For example, both 10 and 20 mg/kg doses increased and decreased BGluM in the cortex and the midbrain of female rats, respectively. On the contrary, other regions showed dose-specific effects such as reduced BGluM in the thalamus by 10 mg/kg ketamine and in the hypothalamus by 20 mg/kg ketamine. It is interesting that a high-dose ketamine infusion decreased BGluM in the hypothalamus of female rats as one would expect increased hypothalamic activity based on the elevation of plasma CORT levels in the animals. However, it may be due to a negative feedback mechanism in the HPA axis regulated by elevated CORT levels from adrenal glands, which suppresses hypothalamic activity [56,57]. It is important to note that the current findings from female rats do not agree with the BGluM results from the previous male rat study [13]. However, that study tested only one dose of ketamine (10 mg/kg) on BGluM in male rats, and furthermore, this may highlight the potential sex-related differences in BGluM following IV ketamine infusion in rodents. Literature on sex-related differences in the effects of ketamine on brain glucose utilization is scarce. A preclinical study using only male rats reported that a lower dose (5 mg/kg) ketamine infusion produced increased BGluM in most brain areas studied, including the cortex, midbrain, and thalamus [58]. In healthy men, ketamine administration before ^18^F-FDG-PET scanning increased glucose metabolic rate in the whole brain, with most prominent increases in the thalamus and frontal and parietal cortices [59]. Clinical studies of healthy men and women receiving ketamine or saline infusions before initiation of ^18^F-FDG-PET scanning reported increased BGluM in the prefrontal cortex [60] and the whole brain, especially in the frontal and cingulate anterior cortices [61], with some correlation between these uptake patterns and psychotic or delusional symptoms of ketamine [60,61]. Taken together, the previous and current findings suggest that subanesthetic doses of ketamine administration may produce sex-specific effects on regional glucose utilization in male and female subjects which may have important clinical implications. 

The current study is not without limitations. First, a compressed timeline was used to investigate the effects of ketamine on fear memory retrieval and fear extinction within three days of IV ketamine infusion. Therefore, caution is required when interpreting the current results to extrapolate the effects of ketamine on long-term fear memory rather than short-term memory. Indeed, it has been shown that long-term fear memory was not significantly affected by post-fear conditioning IV ketamine infusion in male rats [30]. Thus, a further study is required to address the issue of short-term vs. long-term fear memory between male and female animals. Second, saline solution was used as a control infusion in this study while midazolam, a benzodiazepine with amnesic properties, is commonly used as a placebo control in clinical studies on ketamine because midazolam produces similar dissociative symptoms. However, animals do not experience placebo effects, unlike humans, and the animals were tested at least two days after the ketamine infusion, which is well beyond the timeline of IV ketamine-induced dissociation and immediate effects on behavior [62]. 

In conclusion, subanesthetic doses of a single IV ketamine infusion following fear conditioning produced robust effects on locomotor activity, stress hormone levels, fear memory, and regional glucose utilization in adult female rats. Given the heterogeneous effects of IV ketamine on these outcome variables in male and female rats, it is important to include both sexes when investigating the effects of ketamine on fear- and stress-related disorders. Because both men and women are likely to receive ketamine as a trauma analgesic, better understanding of the sex-related differences in the effects of ketamine on trauma-related responses may improve future clinical treatment protocols. 

## 4. Materials and Methods

### 4.1. Animals

Adult female Sprague–Dawley rats (9 weeks old, approximately 200 g upon arrival, Envigo Laboratories, Dublin, VA, USA) were used for the entire study. A jugular venous catheter (Instech, Plymouth Meeting, PA, USA) was surgically implanted under isoflurane anesthesia at Envigo Laboratories (Dublin, VA, USA) prior to animal arrival as previously described [16]. The catheter was tunneled under the skin and connected to a vascular access button (Instech, Plymouth Meeting, PA, USA) that exited dorsally between the rodent scapulae. Animals were singly housed in clear Plexiglas shoebox cages in a climate-controlled environment with food/water available ad libitum. Animals were habituated to a 12-h reversed dark/light cycle (lights off at 0600 and on at 1800 h) and handled daily for one week prior to testing. Catheters were flushed twice per week to maintain catheter patency and locked with 0.2 mL of sterile heparin/glycerol solution (1:1 dilution). All procedures were performed during the dark cycle and were in accordance with the National Institutes of Health Guide for the Care and Use of Laboratory Animals and approved by the Institutional Animal Care and Use Committee at the Uniformed Services University (Bethesda, MD, USA).

### 4.2. Auditory Fear Conditioning

The overall study design is shown in Figure 1A. The fear conditioning experiment was carried out in a chamber (Context A) constructed with Plexiglas and aluminum walls (Coulbourn Instruments, Lehigh Valley, PA, USA) as described previously [13]. After a 180-s acclimation period, rats received three pairings of an auditory tone (5 kHz, 75 dB, 20 s) (conditioned stimulus–CS) that co-terminated with a mild footshock (0.6 mA, 1 s) (unconditioned stimulus–US) at the end of the tone. A variable inter-trial interval (ITI) that ranged from 90 to 120 s was used to prevent tone prediction by the animals. Animals were removed from the chamber 60 s after the last tone-footshock pairing and received an IV infusion of either saline or ketamine.

### 4.3. IV Ketamine Infusion

Racemic (±) ketamine hydrochloride (100 mg/mL) (Mylan Institutional LLC, Rockford, IL, USA) was diluted in 0.9% sterile saline and was administered in infusion chambers (Med Associates Inc., St. Albans, VT, USA) as previously described [13]. Animals were attached to a tether for ketamine infusion, but otherwise had free mobility in the chambers. Spontaneous locomotor activity was monitored by two infrared photobeams within each chamber during the 2-h infusion period. 

Animals were randomly assigned to four groups: saline control (*n* = 16), 2 mg/kg (*n* = 13), 10 mg/kg (*n* = 15), or 20 mg/kg (*n* = 16) ketamine, infused over 2 h. Immediately after fear conditioning, ketamine groups received a ketamine bolus to facilitate ketamine plasma loading before the initiation of the infusion. Ketamine IV bolus doses were 0.5 mg/kg for the 2 mg/kg group and 2 mg/kg for the 10 and 20 mg/kg groups, consistent with the previous male rat study [13]. The saline control group received a saline bolus followed by a 2-h saline infusion. All IV infusions were delivered in a 1 mL/kg/h volume. Immediately after the infusion, blood (0.3 mL) was drawn from the jugular venous catheter and collected in an EDTA tube for stress hormone assays. 

### 4.4. Fear Memory Testing

Cued fear memory retrieval and fear extinction were tested in a novel context (Context B), which was different from the conditioning chamber (Context A). Two days after fear conditioning and ketamine infusion, animals underwent fear memory retrieval followed by fear extinction in a single session (Cue Test 1). After an acclimation period of 3 min, animals were exposed to 12 auditory tone (CS) presentations (5 kHz, 75 dB, 20 s) with an inter-trial interval of 90–120 s in Context B. Freezing during each CS presentation (20 s) was scored and average freezing of two consecutive CS presentations (CS1 and CS2, CS3 and CS4, etc.) was calculated and described as Blocks 1 to 6 in Figure 2A,B. Freezing behavior was defined as an absence of body movement, except as required for respiration. The next day (Day 3), the same test was repeated to determine fear extinction retrieval in the animals (Cue Test 2). On the following day (Day 4), contextual fear and cued fear renewal were tested in Context A. Animals were placed in the fear conditioning chamber (Context A), and freezing to the context was measured for 180 s. Then, a single auditory tone (5 kHz, 75 dB, 20 s) was presented and freezing after the tone was measured for 300 s. Two observers blind to the treatment condition scored the freezing time, and the freezing time was converted to a percent freezing during the testing period.

### 4.5. Estrous Cycle Monitoring

The estrous cycle of female rats was monitored during the acclimation period and on the day of fear conditioning and ketamine infusion using a vaginal cytology method. Vaginal cells (leukocytes and epithelial cells) were collected using a vaginal swab and spread onto microscope slides (Fisher Scientific, Waltham, MA, USA). Subsequently, the vaginal smears were stained with 0.1% crystal violet staining solution (Fisher Scientific, Waltham, MA, USA) to enhance cellular morphology. Based on the prominent cell type on the fear conditioning/ketamine infusion day (Day 0), animals were assigned to one of three groups: estrus (cornified epithelial cells), diestrus (leukocytes), and proestrus (nucleated epithelial cells).

### 4.6. CORT and Progesterone Assays

Plasma CORT and progesterone levels were measured using ELISA kits (Arbor Assays, Ann Arbor, MI, USA) as described previously [29,30]. The blood samples were centrifuged at 4000 rpm for 10 min at 4 °C, and plasma was collected and stored at −40 °C. For the ELISA, a serial dilution of standard samples was prepared and added to a 96-well plate. According to the manufacturer’s protocol, diluted plasma sample, antibody, and CORT or progesterone conjugate were added into each well. The plate was covered with a plastic film and incubated at room temperature on an orbital shaker. After incubation, the plate was washed with wash buffer multiple times. TMB substrate was added to every well, and the plate was incubated for 30 min at room temperature before stop solution was added. The optical density was read at 450 nm using an Infinite 200 Pro Microplate Reader (Tecan US, Morrisville, NC, USA).

### 4.7. ^18^F-FDG-PET and CT Imaging

In a separate experiment, animals were randomly assigned into three groups: saline control (*n* = 11), 10 mg/kg (*n* = 9), or 20 mg/kg (*n* = 12) ketamine. Two ^18^F-FDG-PET and computed tomography (CT) scans were obtained for each animal at baseline, 4 to 5 days before the experiment (scan 1) and immediately after fear conditioning and ketamine infusion (scan 2). ^18^F-FDG-PET and CT images were acquired using an Inveon multimodality preclinical scanner (Siemens Medical Solutions, Erlangen, Germany) in the small animal imaging facility at USU as described previously [13]. Animals were briefly anesthetized with isoflurane (4% induction and 1.5–2.5% maintenance) and injected with 1.7 ± 0.153 mCi (62.9 ± 5.7 MBq) ^18^F-FDG through the tail vein. After ^18^F-FDG injection, the animal was returned to a clean cage during the uptake period (30 min) in a quiet room adjacent to the PET and CT scanner. Animals were undisturbed and exhibited minimal movement in their cages during the ^18^F-FDG uptake period. After uptake, animals were anesthetized with isoflurane (4% induction and 1.5 to 2.5% maintenance) to perform PET and CT scans. Physiologic monitoring during the scan included measurements of temperature, respiration rate, heart rate, and oxygen saturation.

### 4.8. ^18^F-FDG-PET/CT Data Analysis

Image processing and analysis of the ^18^F-FDG-PET/CT data were performed using VivoQuant software (ver 3.0, inviCRO, Boston, MA, USA) as described previously [13]. ^18^F-FDG-PET data were resampled to match CT voxel size (0.22-mm^3^ isotropic) and dimensions (384 × 384 × 425). The PET data were converted to units of activity (μCi) and registered to the CT image. Co-registered PET and CT images were uniformly cropped to a region surrounding the brain (170 × 170 × 240) and automatically registered to a three-dimensional rat brain atlas using an algorithm that combines a rigid transformation of the data and scaling of the atlas. The 13 brain regions included basal ganglia, thalamus, amygdala, cerebellum, cortex, hypothalamus, midbrain, corpus callosum, olfactory bulbs, hippocampus, septal area, white matter, and ventricles. The standard uptake value (SUV) of each region was normalized with the SUV of the entire brain atlas (whole-brain normalization) of the same animal to reduce between-subject variability.

### 4.9. Statistics

All data are presented as mean ± standard error of the mean (SEM) and are normally distributed with similar variances across the groups by the D’Agostino and Pearson normality test. A two-way repeated measures (RM) analysis of variance (ANOVA) with ketamine and time as independent variables was used to analyze locomotor activity, fear memory, and BGluM data. Holm–Sidak’s post-hoc tests were used to compare group differences following the two-way RM ANOVA. One-way ANOVA was used for cell type data analysis in ketamine groups. Sample sizes for stress hormone data (*n* = 13–14 per group) are smaller than for behavioral data (*n* = 13–16 per group) due to difficulty of catheter blood draw in some of the animals. Data were analyzed using GraphPad Prism (GraphPad Software, version 9.0) and the accepted level of significance was *p* < 0.05.

## Data Availability

Data are available upon reasonable request to the corresponding author.
